# Knowledge and Awareness of Myopia Among Parents and Teachers of Schoolchildren Aged 6–15 Years in Beirut, Lebanon

**DOI:** 10.3390/vision10010011

**Published:** 2026-02-12

**Authors:** Ameer Abou Adela, Vanessa R. Moodley, Yazan Gammoh

**Affiliations:** 1 School of Health Sciences, Discipline of Optometry, University of KwaZulu Natal (UKZN), Private Bag X54001, Durban 4000, South Africa; moodleyvr@ukzn.ac.za; 2 Department of Optometry, Faculty of Allied Medical Sciences, Al-Ahliyya Amman University, Amman 19111, Jordan; y.gammoh@ammanu.edu.jo

**Keywords:** myopia, parents, teachers, awareness, Beirut, Lebanon, children

## Abstract

Background: Using a cross-sectional design, this study assessed and compared myopia knowledge among parents and teachers of schoolchildren aged 6–15 years in Beirut, Lebanon. Methods: Two cross-sectional surveys were conducted between October 2022 and February 2024 among parents (*n* = 1256) and teachers (*n* = 366) of children aged 6–15 years. Using validated online Google Form questionnaires, data were collected on demographics, awareness, risk factors, and myopia knowledge, and analyzed with Statistical Package for the Social Sciences version 28 (SPSS v28) through descriptive statistics and logistic regression. Results: Findings showed that 78.3% of parents and 79.5% of teachers had poor knowledge of myopia. Among teachers, better knowledge was linked to being male, having a family history of myopia, positive attitudes toward eyeglasses use, and attending regular or occasional eye care visits (all statistically significant). Among parents, higher knowledge was associated with having previously heard of myopia, higher income and education levels, and a family history of myopia, while parents of private-school children were less knowledgeable. Odds ratios below 1 indicate lower odds of good myopia knowledge relative to the reference category. Conclusions: Both groups showed inadequate knowledge, underscoring the urgent need for targeted educational interventions to improve myopia awareness and prevention.

## 1. Introduction

Myopia is a common refractive error in which distant objects appear blurry while near vision remains clear [1]. It results from axial elongation of the eyeball or increased corneal curvature and is now recognized as a major global public health issue, particularly among children and adolescents [2]. The World Health Organization (WHO) estimates that by 2050, myopia will affect nearly 50% of the global population [3]. This rise is linked to behavioral and environmental factors such as increased near work, prolonged screen use, reduced outdoor activity, and limited exposure to natural light during childhood. Without proper management, myopia can negatively impact academic performance, social interactions, and overall quality of life [4] and may lead to complications, including retinal detachment and myopic macular degeneration [5].

Parents significantly influence children’s health-seeking behavior, treatment compliance, and adoption of preventive practices [6]. However, parental awareness of myopia varies across regions. In China, a survey of 2500 parents showed that although 55.1% recognized myopia as a medical condition, over 70% were unaware of its potential complications [7,8]. Tao et al. (2022) also reported that children of parents with lower educational levels and higher myopic spherical equivalence experienced faster myopia progression, highlighting the need for targeted interventions [9]. In Singapore, 57% of parents correctly identified myopia in children, yet many underestimated the impact of device use and lacked knowledge of preventive measures.

Teachers, as daily observers of children, are often the first to detect potential vision issues. Their knowledge can support early referral and help reinforce healthy visual habits in the classroom [6]. However, teacher awareness also varies. In Mysore, India, although 80% of primary school teachers were aware of refractive errors, none had experience identifying visual problems in students [10].

Despite the importance of both groups, few studies have compared myopia knowledge between parents and teachers within the same population. In Lebanon, myopia prevalence among schoolchildren in Beirut has been reported at 14.22% [11], and UNICEF has highlighted major gaps in health education, including eye health [12]. Given Beirut’s diverse educational environment and uneven access to healthcare, assessing the knowledge of parents and teachers is essential for effective intervention planning.

This study aimed to evaluate and compare myopia knowledge among parents and teachers of children aged 6–15 years in Beirut, Lebanon, focusing on their understanding of causes, symptoms, preventive measures, complications, and treatment options.

## 2. Materials and Methods

This study used a quantitative, cross-sectional, descriptive design to assess myopia awareness and knowledge among two distinct groups: parents of children aged 6–15 years and teachers instructing this age group in public, private, and semi-private schools (private schools partially supported by the government) in Beirut, Lebanon. The study was conducted between October 2022 and February 2024 in Beirut, Lebanon. Ethical approval for this study was obtained from the University of KwaZulu-Natal Biomedical Research Ethics Committee (BREC/00004450/2022) and the Institutional Review Board of the American University of Science and Technology (AUST-IRB-20220822). Informed consent was obtained from all subjects involved in the study. All participants’ identities were anonymized, and data confidentiality was strictly maintained throughout the study in accordance with the Declaration of Helsinki.

### 2.1. Study Setting and Participants Characteristics

A stratified cluster random sampling technique was employed. Beirut’s school network was divided into three geographic clusters according to the Ministry of Education, containing 30, 88, and 25 schools, respectively. Within each cluster, schools were randomly selected to reflect private, public, and semi-private institutions and the socioeconomic diversity of the city. In selected schools, every third student on the class list from grades 1 to 9 was chosen, alternating by sex to ensure proportionality. The starting point for selection of every third student was randomly assigned to avoid convenience sampling within classes. The sample sizes for both parents and teachers were determined to ensure statistical validity and representativeness. For the parent group, the required sample was calculated using Daniel’s formula (Available online: https://www.scirp.org/reference/referencespapers?referenceid=396450 (accessed on 13 June 2024)), assuming a 50% prevalence, 95% confidence level (Z = 1.96), and 5% margin of error. Based on a population of 3564 students, a minimum of 351 parent responses was needed. Parents of the 3564 selected students were invited to participate, of whom 1256 consented and completed the questionnaire.

This yielded a 35.2 percent response rate. This level of participation is acceptable but may introduce nonresponse bias, as parents who chose to respond could differ in interest or awareness compared to non-respondents. Additionally, the use of self-administered questionnaires introduces self-report bias, including possible inaccuracies due to recall or the desire to provide socially acceptable answers. These factors should be considered when interpreting the findings.

For the teacher sample, schools with fewer than 30 teachers included all staff, while those with larger numbers used 30% random sampling. The estimated population was 756 across selected schools and applying a similar approach, the minimum required sample size was 254. The structured online questionnaire was completed by 366 teachers, exceeding the minimum threshold for reliable analysis. This method ensured a representative sample that reflected the socioeconomic and demographic diversity of the population.

### 2.2. Study Tools

Two structured, validated questionnaires (one for each group) were administered through Google Forms. Each consisted of demographic items (sex, place of residence, type of school, and nationality) and knowledge questions regarding myopia’s causes, complications, prevention strategies, and treatment options. Both groups were also asked about their awareness of myopia, sources of information, and whether they or their respective family members have myopia. The survey also explored beliefs about the impact of reading, electronic device use, genetics, and nutrition on myopia. Additionally, it examined their attitudes toward eyeglasses and habits concerning regular eye check-ups. Responses were closed-ended (“yes”, “no”, or “I don’t know”).

The questionnaires were newly developed for this study and underwent content validation by experts, pilot testing (*n* = 20), and principal component analysis (PCA) to support construct validity, with the extracted components explaining an acceptable proportion of the total variance. Internal consistency was satisfactory, with a Cronbach’s alpha of 0.7. All data were securely stored in a password-protected system to maintain confidentiality.

### 2.3. Statistical Analysis

Data was exported from Google Forms and Microsoft Excel for initial cleaning and coding. Statistical analyses were performed using IBM SPSS V28. Demographics and knowledge responses were summarized using frequencies and percentages, then tested associations with chi-square and logistic regression for both parents and teachers.

Knowledge scores were categorized as “good” or “poor” based on performance thresholds established during questionnaire validation (e.g., correctly answering more than 50% of knowledge items). Chi-square (χ^2^) tests were used to examine associations between categorical demographic or behavioral variables (e.g., sex, education, income, eye-care visits) and overall knowledge level. The 50% threshold was selected a priori to allow comparability with similar knowledge-based surveys and was supported by sensitivity analyses using alternative cut-offs, which yielded consistent associations.

Binary logistic regression analysis was conducted to identify independent predictors of good myopia knowledge in each group. Variables with a *p*-value < 0.25 in bivariate analyses were entered into the regression model using the stepwise selection method. Prior to modeling, multicollinearity was assessed using variance inflation factors (VIFs). Model fit was evaluated using the Hosmer–Lemeshow goodness-of-fit test. Odds ratios (ORs) with 95% confidence intervals (CIs) and *p*-values were reported to determine the strength and significance of associations. A *p*-value < 0.05 was considered statistically significant for all analyses. Although schools were sampled using a cluster design, regression analyses were performed at the individual level; clustering by school was therefore accounted for in the interpretation of results and included as a limitation.

## 3. Results

The demographic profile of the study participants reflects a predominantly Lebanese population of parents and teachers living mainly in Beirut. Among the 1256 parents, most were mothers, followed by fathers, with only a small percentage representing siblings or other guardians. The majority were Lebanese nationals, resided in Beirut, and had children enrolled in private schools. Educational levels varied, with only one-quarter holding university degrees and fewer than 10% possessing postgraduate qualifications. Economically, over one-fifth were unemployed, while a smaller segment reported higher monthly incomes above 6,000,000 L.L. The teacher sample (*n* = 366) showed a similar demographic pattern, with a high proportion of female and Lebanese participants, most of whom also lived in Beirut. Their employment was nearly balanced between private and public schools, with only a small minority working in semi-private settings (Table 1).

Teachers showed high personal and family exposure to myopia, with two-thirds reporting a myopic family member and over 40% being myopic themselves. Among parents, general awareness was moderate, yet meaningful knowledge remained limited: although nearly two-thirds had heard of myopia, only about one-third visited eye care professionals regularly, and just one-fifth showed good knowledge based on the assessment items. Teachers were more aware of myopia than parents overall, but their depth of knowledge was similarly low, with only 20.5% classified as having good understanding. Among eyeglasses users, most wore their glasses consistently, and almost all reported that their vision improved with use (Table 2).

Table 3 reveals notable knowledge gaps among parents and teachers. While over half recognized the role of genetics (58.1% of parents; 75.1% of teachers) and around two-thirds identified screen use as a cause, understanding of complications was limited. Only about one in five knew that myopia could lead to retinal detachment (22.0%), and fewer than 30% recognized vision impairment as a potential outcome. Preventive knowledge was similarly low, with only around 19% identifying outdoor time as protective and less than half linking reduced screen use to prevention. Awareness of advanced treatment options such as atropine drops, orthokeratology, and multifocal lenses was especially limited, with more than 60% responding “I don’t know.”

Based on Table 4, effect-size estimates indicated that several predictors had a meaningful influence on good myopia knowledge among both teachers and parents.

Among teachers, sex and attitudes toward eyeglasses were important predictors. Female teachers, compared with male teachers, had lower odds of good knowledge (OR = 0.36, 95% CI: 0.17–0.76), indicating that they were less likely to demonstrate good myopia knowledge relative to males. Teachers’ attitudes toward eyeglass use were significantly associated with myopia knowledge levels (OR = 0.35, 95% CI: 0.14–0.89), indicating variation in knowledge patterns across attitudinal groups. Uncertainty about one’s own myopia status (“I don’t know” vs. yes) showed the largest effect (OR = 3.97, 95% CI: 1.73–9.13), indicating a substantially higher likelihood of good knowledge. The associated confidence intervals confirm that these effects are statistically significant and meaningful in magnitude.

Among parents, school type, education, income, and family history were significantly associated with good myopia knowledge (Table 4). Parents of learners attending private schools had lower odds of good knowledge compared with those in official schools (OR = 0.61, 95% CI: 0.42–0.88). Lower educational attainment was also associated with reduced odds of good knowledge when compared with postgraduate education (primary vs. postgraduate: OR = 0.86, 95% CI: 0.71–0.90; no schooling vs. postgraduate: OR = 0.71, 95% CI: 0.68–0.87). Higher household income (>6,000,000 L.L.) was associated with lower odds of good knowledge compared with lower income levels (OR = 0.48, 95% CI: 0.26–0.89). In addition, family history of myopia showed significant associations with good knowledge, with both “yes” and “I don’t know” responses differing from the reference category (Table 4). A forest plot of adjusted odds ratios with 95% confidence intervals is provided in the Supplementary Materials (Supplementary Figure S1).

Results (Table 5) showed that knowledge of myopia varied noticeably across different demographic and experiential factors. Overall, parents and teachers had similar knowledge levels, though a slightly higher proportion of parents showed good knowledge (20.8% vs. 19.1% among teachers). This difference was small and only borderline significant (*p* = 0.052).

Awareness strongly influenced knowledge. Among participants who had previously heard of myopia, 25.2% showed good knowledge compared with only 15.1% of those who had not (*p* < 0.001). Regular interaction with eye care professionals was another important factor: nearly 29% of respondents who visited eye care providers regularly had good knowledge, compared with 21.7% of those who visited occasionally and just 15.1% of those who never sought care (*p* < 0.001).

Personal and family experience with myopia also played a clear role. Respondents who were myopic themselves showed better knowledge (26.9%) than those who were not (19.3%) or were unsure (17.5%) (*p* < 0.001). A similar pattern was seen with family history: participants with at least one myopic family member had higher knowledge (26.3%) than those without (16.5%) or those who were uncertain (10.8%) (*p* < 0.001).

Attitudes toward eyeglasses were also linked to knowledge. Individuals with a positive attitude toward eyeglasses wearers were more likely to demonstrate good knowledge (23.8%) than those with neutral or negative attitudes (14.6%) (*p* < 0.001).

Binary logistic regression (Table 6) showed several meaningful predictors of myopia knowledge. Teachers were significantly less likely to have good knowledge compared to parents (OR = 0.74, 95% CI: 0.55–0.99, *p* = 0.043), indicating lower school-based awareness. Participants who had not heard about myopia were also less knowledgeable (OR = 0.70, 95% CI: 0.52–0.93, *p* = 0.015), and those who did not attend regular eye-care visits had the strongest reduction in knowledge (OR = 0.52, 95% CI: 0.38–0.72, *p* < 0.001). These effect sizes and confidence intervals highlight the importance of exposure to information and consistent interaction with eye-care professionals.

Attitudinal and family factors also showed meaningful effects. Participants lacking a positive attitude toward eyeglass wear (OR = 0.70, 95% CI: 0.49–0.98, *p* = 0.036) and those unsure about having a family history of myopia (OR = 0.42, 95% CI: 0.25–0.69, *p* = 0.001) were significantly less likely to have good knowledge. Confidence intervals for nonsignificant variables, such as personal myopia status (*p* > 0.05), crossed unity, confirming that personal experience alone did not meaningfully influence knowledge. A forest plot of adjusted odds ratios with 95% confidence intervals is provided in the Supplementary Materials (Supplementary Figure S1).

**Figure 1:**
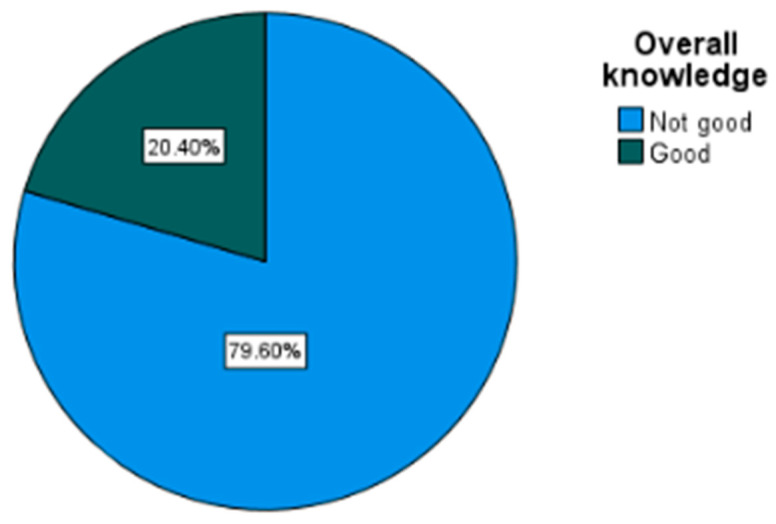
Distribution of overall knowledge about myopia among participants. (%) Percentage.

As shown in Figure 1, the majority showed poor knowledge (79.6%), while only 20.4% showed good knowledge. This indicates that four out of every five respondents lacked sufficient understanding of myopia, its causes, and prevention strategies. The finding aligns with the descriptive analysis, emphasizing that limited awareness remains a major challenge within the studied population.

### Relationship

Table 7, showing the relationship between the participants (parents versus teachers) and overall knowledge of myopia, reveals that 80.9% of teachers and 79.8% of parents showed poor knowledge, while only 19.1% of teachers and 20.2% of parents showed good knowledge. The difference between the two groups was not statistically significant (*p* = 0.231).

## 4. Discussion

This study provides a comprehensive assessment of myopia knowledge among two key stakeholder groups, parents and teachers, of children aged 6 to 15 years in Beirut, Lebanon. The findings reveal that both groups showed generally low levels of knowledge, with 78.3% of parents and 79.5% of teachers categorized as having “poor” knowledge. This widespread lack of awareness is concerning, given the increasing prevalence of myopia globally and the essential roles these groups play in early detection and management.

Among teachers, gender was a significant predictor of knowledge, with male teachers demonstrating higher knowledge scores than females (OR = 0.36, *p* = 0.007). This difference indicates a potential inconsistency in how eye-health information is accessed or integrated across the teaching workforce. Strengthening teacher training with structured eye-health content (basic vision science, signs of myopia, classroom strategies) could promote more uniform knowledge among teachers regardless of background.

Teachers who were unsure of their own myopia status also showed lower knowledge levels, highlighting the role of personal health awareness in shaping professional understanding. In addition, positive attitudes toward eyeglasses and regular visits to eye-care practitioners were associated with better knowledge, consistent with findings from similar teacher-based studies in South Africa and New York [13,14,15,16]. In Beirut, broader barriers continue to limit engagement with routine eye examinations. These include financial pressures, the perception that preventive care is non-urgent, the absence of national vision-screening programs, and the concentration of optometry services in private urban centers. The migration of eye-care professionals has further reduced service availability and increased waiting times. Together, these system-level and economic factors contribute to limited access and irregular use of preventive eye-care services among the community.

Among parents, several sociodemographic factors significantly influenced knowledge. Lower levels of education, lower household income, and affiliation with private schools were associated with poorer knowledge, consistent with international evidence indicating that socioeconomic status is a strong predictor of health literacy [17,18,19]. This finding likely reflects disparities in access to health information and care. Parents with lower education and income may have fewer opportunities to learn about myopia or seek regular eye exams, limiting their exposure to professional advice [20]. This association may reflect contextual factors in Beirut, where competing financial demands and variability in school-based health education could influence healthcare-seeking behavior. Together, these factors explain why socioeconomic status strongly predicts parental knowledge. Parents who had previously heard of myopia, had a family history of the condition, or visited eye care practitioners regularly were more likely to be knowledgeable. These results reinforce the importance of repeated exposure to eye health information through both clinical and social channels.

Despite both groups reporting relatively high awareness of myopia as a term, the specific knowledge regarding its causes, prevention, and complications was lacking. Both groups showed confusion regarding interventions used to control myopia, such as orthokeratology and atropine drops, and few correctly identified serious complications such as retinal detachment or vision impairment [21]. Similar gaps in public understanding have been observed across other health conditions, underscoring the broader need for structured school-based health education. In Beirut, parents and teachers can obtain information on myopia through multiple community-based and professional channels. The Ministry of Public Health, in collaboration with the Ministry of Education, could integrate eye-health awareness into school programs through pamphlets, annual screening days, and optometrist-led outreach. Public education campaigns, such as short TV or social media advertisements, would also help reach a wider audience, especially among parents. Moreover, optometrists can play an active role by conducting school outreach sessions using visual materials and short educational videos to demonstrate early signs of myopia, prevention strategies, and the importance of regular eye exams [22]. Such initiatives would foster collaboration between schools, healthcare providers, and public health authorities, promoting sustained awareness and preventive eye care behaviors.

The rise in digital-device overuse, particularly during and after the COVID-19 lockdowns, has contributed to a notable shift in visual behaviors among children and adolescents. Prolonged online learning, increased screen time, and reduced outdoor activities during periods of restricted movement created conditions that are well recognized as risk factors for myopia onset and progression. The lockdown environment accelerated habits such as close-range viewing and extended near work, while simultaneously limiting exposure to natural light, an important protective factor against myopia. Even after schools reopened, many of these digital habits persisted, reinforcing patterns of excessive screen use. These behavioral changes highlight the need for stronger public-health messaging, digital-use guidelines for families, and school-based strategies to balance screen time with outdoor activities to mitigate the long-term visual impact of pandemic-era routines [23].

The apparent variation in knowledge patterns, for instance, parents being more informed about complications and teachers more familiar with corrective methods, may reflect differences in lived experience versus professional exposure. Teachers may encounter vision issues more often in the classroom, while parents engage with the condition more intimately through their children’s diagnoses and treatment.

The absence of national vision screening programs or public education initiatives on myopia in Lebanon likely contributes to the low knowledge levels observed. A United Nations Children’s Fund (UNICEF) Lebanon report highlighted systemic gaps in school health education, including visual health, a deficiency this study further supports [12]. A recent study conducted in Jordan showed that teacher-led vision screening is a feasible way to detect refractive errors, which could be useful for Lebanon [24]. Currently, school vision screenings in Beirut are sporadic and largely depend on short-term initiatives led by NGOs, hospitals, or university optometry programs. They mainly assess distance visual acuity using a Snellen chart, with limited follow-up or data tracking, leaving many children unscreened or untreated. Based on these findings and the broader context, transforming school screenings into a nationally coordinated, mandatory annual program integrated within the school health system is strongly recommended. This should include a standardized protocol, digital referral tracking, and partnerships with local optometrists for immediate follow-up care. The Ministry of Health and Education should jointly implement an initiative, ensuring that every child identified with vision problems receives corrective glasses within days. Public awareness campaigns, community outreach, and low-cost eyewear subsidies would further strengthen the program, making vision care an essential and accessible part of every child’s educational journey in Lebanon.

## 5. Conclusions

This study reveals that both parents and teachers in Beirut City, Lebanon, exhibited limited understanding of myopia. Addressing these gaps through integrated awareness programs could enhance early detection and management of childhood myopia. Educational efforts must be tailored to engage both stakeholder groups and adapt to local socioeconomic realities.

Taken together, the findings call for coordinated awareness and preventive initiatives targeting both schools and families. Educational interventions should be adapted to each group’s informational needs and practical barriers, involving eye-care professionals and school health systems in their design and delivery [24].

Based on the current gaps identified in Beirut, it is strongly recommended to establish a national school-based myopia program that ensures annual vision screening, structured referral pathways, and affordable access to corrective eyewear. Such a program should be supported by public-health campaigns delivered through social media and television to promote early detection and regular eye examinations.

Additionally, implementing a Teacher Vision Observation Checklist in schools is recommended to help educators identify early signs of visual difficulties among students. This tool enables teachers to observe and report behaviors such as squinting, frequent eye rubbing, difficulty reading from the board, or reduced attention during visual tasks. Regular use of such checklists would support early detection and timely referral, improving both academic performance and eye-health outcomes. Evidence from Kenya, Nigeria, and the United States demonstrates the effectiveness of teacher-led vision observation initiatives, which have improved detection accuracy, increased successful referrals, and even enhanced academic outcomes [25,26,27]. These results align with recommendations from the International Agency for the Prevention of Blindness (2024) [28], which endorses teacher-based observation tools as part of comprehensive school eye-health strategies.

To create a sustainable model that links awareness, early intervention, and equitable access to care, collaboration between the Ministry of Health, Ministry of Education, NGOs such as the Vision Care Association, and local optometrists is essential. Such partnerships would represent an important step toward improving child eye health and reducing the long-term burden of myopia in Lebanon. Future longitudinal and interventional studies should evaluate whether targeted health-education campaigns improve parental and teacher knowledge.

## Figures and Tables

**Table 1:** Demographic profile of parents and teachers.

Demographic Variables		Parents N (%)	Teachers N (%)
Child’s age group (in years)	Between 11 and 15	702 (55.9)	
	Between 6 and 10	554 (44.1)	
Gender	Female	704 (56.1)	315 (86.1)
	Male	552 (43.9)	51 (13.9)
School Type	Private	818 (65.1)	179 (48.9)
	Official	354 (28.2)	174 (47.5)
	Semi-Private	84 (6.7)	13 (3.6%)
Residence	Beirut	1168 (93.0)	238 (65.0)
	Outside Beirut	88 (7.0)	128 (35.0)
Nationality	Lebanese	964 (76.8)	352 (96.2)
	Non-Lebanese	292 (23.2)	14 (3.8)
Education Level	No Formal Education	74 (5.9)	
	Primary	288 (22.9)	
	Secondary	390 (31.1)	
	University Degree	318 (25.3)	
	Postgraduate	94 (7.5)	
Household income (in Lebanese Lira L.L.)	> 6,000,000 L.L.	214 (17.0)	
	≤6,000,000 L.L.	626 (49.9)	
	Unemployed	266 (21.2)	
	Not reported	150 (11.9)	

(%) Percentage, (N) Number.

**Table 2:** Myopia awareness, attitude toward eyeglasses, and eye care visits.

Variable	Responses	Parents N (%)	Teachers N (%)
**Myopia awareness**			
Have you ever heard about myopia?	Yes	802 (63.9)	291 (79.5)
	No	454 (36.1)	75 (20.5)
Do you have myopia (poor distance vision)?	I do not know	258 (20.5)	50 (13.7)
	No	530 (42.2)	164 (44.8)
	Yes	468 (37.3)	152 (41.5)
Do you have any family members with myopia (poor distance vision)?	I do not know	178 (14.2)	26 (7.1)
	No	312 (24.8)	99 (27.0)
	Yes	766 (61.0)	241(65.8)
**Attitude toward eyeglasses**			
Do you have a positive attitude towards individuals who wear eyeglasses?	Yes	976 (77.7)	304 (83.1)
	No	280 (22.3)	62 (16.9)
**Frequency of eyecare visits**			
How frequently do you visit an eye care practitioner (Optometrist or Ophthalmologist)?	Yes	434 (34.6)	182 (49.7)
	Sometimes	284 (22.6)	98 (26.8)
	No	538 (42.8)	86 (23.5)

(%) Percentage, (N) Number.

**Table 3:** Knowledge of myopia: corrective options, preventive measures, causes, and complications.

Knowledge Domain	Item/Question	Responses	Parents N (%)	Teachers N (%)
Corrective Measures	Does wearing eyeglasses correct myopia?	Yes	668 (53.2)	214 (58.5)
		No	224 (17.8)	74 (20.2)
		I don’t know	364 (29.0)	78 (21.3)
	Does wearing contact lenses correct myopia?	Yes	396 (31.5)	138 (37.7)
		No	378 (30.1)	93 (25.4)
		I don’t know	482 (38.4)	135 (36.9)
	Does surgery treat myopia?	Yes	642 (51.1)	217 (59.3)
		No	142 (11.3)	29 (7.9)
		I don’t know	472 (37.6)	120 (32.8)
Preventive Measures	Does less use of electronic devices help in preventing myopia?	Yes	654 (52.1)	189 (51.6)
		No	250 (19.9)	71 (19.4)
		I don’t know	352 (28.0)	106 (29.0)
	Do outdoor activities prevent myopia onset?	Yes	244 (19.4)	70 (19.1)
		No	424 (33.8)	124 (33.9)
		I don’t know	588 (46.8)	172 (47)
	Does looking at far objects prevent myopia?	Yes	384 (30.6)	108 (29.5)
		No	294 (23.4)	81 (22.1)
		I don’t know	578 (46.0)	177 (48.4)
	Do regular breaks help prevent myopia?	Yes	594 (47.3)	154 (42.1)
		No	152 (12.1)	46 (12.6)
		I don’t know	310 (40.6)	166 (45.4)
	Do annual eye check-ups prevent myopia progression?	Yes	638 (50.8)	189 (51.6)
		No	252 (20.1)	66 (18.0)
		I don’t know	366 (29.1)	111 (30.3)
	Does using atropine eye drops prevent or slow myopia?	Yes	228 (18.2)	60 (16.4)
		No	256 (20.4)	61 (16.7)
		I don’t know	772 (61.5)	245 (66.9)
	Do multifocal glasses prevent myopia progression?	Yes	316 (25.2)	138 (37.7)
		No	204 (16.2)	29 (7.9)
		I don’t know	736 (58.6)	199 (54.4)
	Do orthokeratology lenses prevent myopia progression?	Yes	146 (11.6)	47 (12.8)
		No	284 (22.6)	62 (16.9)
		I don’t know	826 (65.8)	257 (70.2)
Causes	Does frequent use of electronic devices cause myopia?	Yes	830 (66.1)	240 (65.6)
		No	134 (10.7)	
		I don’t know	292 (23.2)	
	Do genetic factors cause myopia?	Yes	730 (58.1)	275 (75.1)
		No	110 (8.8)	
		I don’t know	416 (33.1)	
	Does frequent reading cause myopia?	Yes	374 (29.8)	128 (35.0)
		No	344 (27.4)	
		I don’t know	538 (42.8)	
	Does healthy nutrition help in preventing myopia?	Yes	336 (26.8)	93 (25.4)
		No	252 (20.1)	109 (29.8)
		I don’t know	668 (53.2)	164 (44.8)
Complications	Is cataract a complication of myopia?	Yes	186 (14.8)	43 (11.7)
		No	166 (13.2)	32 (8.7)
		I don’t know	904 (72.0)	291 (79.5)
	Is glaucoma a complication of myopia?	Yes	174 (13.9)	45 (12.3)
		No	142 (11.3)	35 (9.6)
		I don’t know	940 (74.8)	286 (78.1)
	Is retinal detachment a complication of myopia?	Yes	276 (22.0)	56 (15.3)
		No	158 (12.6)	36 (9.8)
		I don’t know	822 (65.4)	274 (74.9)
	Is vision impairment a complication of myopia?	Yes	350 (27.9)	68 (18.6)
		No	292 (23.2)	90 (24.6)
		I don’t know	614 (48.9)	208 (56.8)

(%) Percentage, (N) Number.

**Table 4:** Factors affecting myopia knowledge in parents and teachers.

Factor	Comparison Group	OR	95% CI	*p*-Value	Sample
Sex	Female vs. Male	0.36	0.17–0.76	*p* = 0.007	Teachers
Uncertainty about myopia status	“I don’t know” vs. Yes	3.97	1.73–9.13	*p* = 0.001	
Family history of myopia	Yes vs. “I don’t know”	0.10	0.02–0.52	*p* = 0.006	
Attitude toward eyeglasses	Positive vs. Negative	0.35	0.14–0.89	*p* = 0.027	
Eyecare visits (sometimes)	Sometimes vs. Yes	0.40	0.19–0.87	*p* = 0.021	
School type	Private vs. Official	0.61	0.42–0.88	*p* = 0.008	Parents
Education level	Primary vs. Postgraduate	0.86	0.71–0.90	*p* = 0.041	
	No schooling vs. Postgraduate	0.71	0.68–0.87	*p* = 0.014	
Income	>6,000,000 L.L. vs. ≤6,000,000 L.L.	0.48	0.26–0.89	*p* = 0.019	
Myopia awareness	Yes, vs. No	0.56	0.38–0.83	*p* = 0.003	
Eyecare visits	Yes vs. No	0.81	0.57–1.17	*p* = 0.268	
Family history of myopia	Yes vs. No	0.65	0.44–0.98	*p* = 0.038	
	“I don’t know” vs. Yes	0.44	0.25–0.77	*p* = 0.004	

(%) Percentage, (N) Number, (OR) Odds Ratio, (CI) Confidence Interval.

**Table 5:** Association between demographic and behavioral factors and overall knowledge about myopia.

		Overall Knowledge				Chi-Square *p*-Value
		Poor		Good		
		Count	%	Count	%	
Type	Teachers	317	80.9	75	19.1	*p* = 0.052
	Parents	1068	79.2	280	20.8	
Have you ever heard about myopia?	No	449	84.9	80	15.1	*p* < 0.001
	Yes	818	74.8	275	25.2	
Have you visited any Optometrist, Ophthalmologist or eyecare practitioner on regular basis?	No	530	84.9	94	15.1	*p* < 0.001
	Sometimes	299	78.3	83	21.7	
	Yes	438	71.1	178	28.9	
Do you have myopia (poor distance vision)?	I don’t know	254	82.5	54	17.5	*p* < 0.001
	No	560	80.7	134	19.3	
	Yes	453	73.1	167	26.9	
Do you have any family members with myopia (poor distance vision)?	I don’t know	182	89.2	22	10.8	*p* < 0.001
	No	343	83.5	68	16.5	
	Yes	742	73.7	265	26.3	
Do you have positive attitude towards the person wearing eyeglasses?	No	292	85.4	50	14.6	*p* < 0.001
	Yes	975	76.2	305	23.8	

(%) Percentage.

**Table 6:** Binary logistic regression analysis of factors associated with overall knowledge about myopia.

		OR(B)	95% CI for EXP (B)		*p*-Value
			Lower	Upper	
Type	Teachers’ vs. Parent	0.737	0.548	0.991	*p* = 0.043
Have you ever heard about myopia?	No vs. Yes	0.695	0.518	0.932	*p* = 0.015
Have you visited any Optometrist, Ophthalmologist or eyecare practitioner on regular basis?	No vs. Yes	0.523	0.379	0.723	*p* < 0.001
Have you visited any Optometrist, Ophthalmologist or eyecare practitioner on regular basis?	Sometimes vs. Yes	0.726	0.533	0.988	*p* = 0.041
Do you have positive attitude towards the person wearing eyeglasses?	No vs. Yes	0.695	0.494	0.977	*p* = 0.036
Do you have any family members with myopia (poor distance vision)?	I don’t know vs. Yes	0.415	0.251	0.687	*p* = 0.001
Do you have any family members with myopia (poor distance vision)?	No vs. Yes	0.762	0.551	1.052	*p* = 0.098
Do you have myopia (poor distance vision)?	I don’t know vs. Yes	1.137	0.765	1.691	*p* = 0.525
Do you have myopia (poor distance vision)?	No vs. Yes	0.931	0.699	1.241	*p* = 0.627

(%) Percentage, (OR) Odds Ratio, (CI) Confidence Interval.

**Table 7:** Distribution of overall knowledge on myopia among parents and teachers.

Study Group	Overall, Knowledge				*p*-Value
	Poor		Good		
	Count	%	Count	%	
Parents	1068	79.82	280	20.18	*p* = 0.231
Teachers	317	80.90	75	19.10	

(%) Percentage.

## Data Availability

The data set used and/or analyzed during the current study is available from the corresponding author on reasonable request due to the privacy of the volunteers.

## Supplementary Materials

The following supporting information can be downloaded at: https://www.mdpi.com/article/10.3390/vision10010011/s1, Figure S1: Forest plot of adjusted odds (ORs) for good myopia knowledge.

## Abbreviations

The following abbreviations are used in this manuscript:

UKZN	University of Kwazulu-Natal
AUST	American University of Science and Technology
UNICEF	United Nations Children’s Fund
WHO	World Health Organization
PCA	Principal Component Analysis
ORs	Odds Ratios
CIs	Confidence Intervals
χ^2^	Chi-square
SPSS	Statistical Package for the Social Sciences
VCA	Vision Care Association
BASD	Beirut Association for Social Development
