# Supplementary material for: Knowledge and Awareness of Myopia Among Parents and Teachers of Schoolchildren Aged 6–15 Years in Beirut, Lebanon
Source: Vision (Basel). 2026 Feb 12;10(1):11. doi: 10.3390/vision10010011 (PMC12922107; doi:10.3390/vision10010011)
Supplement: Supplementary file 1 [file vision-10-00011-s001.zip › vision-3977794-SI.pdf]

Supplementary Material

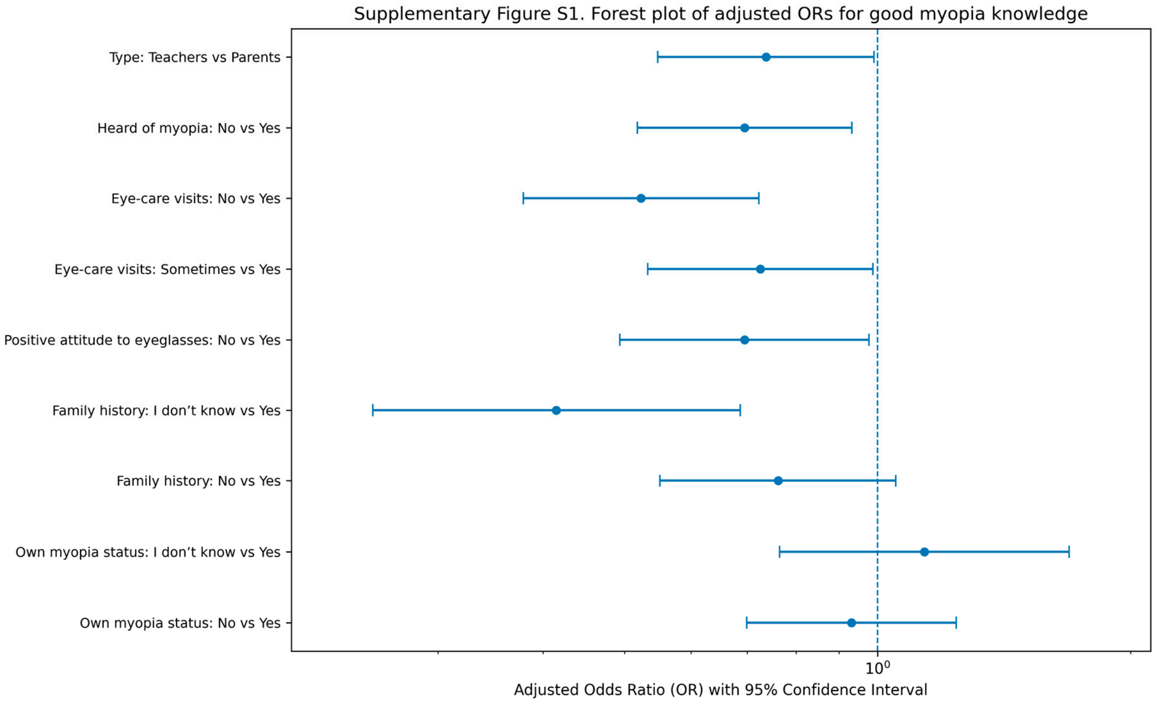

Figure S1. Forest plot of adjusted odds ratios (ORs) with 95% confidence intervals for factors associated with good myopia knowledge among parents and teachers in Beirut, Lebanon. The vertical dashed line represents the null value (OR = 1).
